# 27 Single center inception cohort of enthesitis-related arthritis: spectrum of characteristics, course and treatment approaches

**DOI:** 10.1093/rheumatology/keac496.023

**Published:** 2022-10-07

**Authors:** Fatma Gül Demirkan, Figen Çakmak, Nuray Aktay Ayaz

**Affiliations:** Department of Pediatric Rheumatology, Istanbul Faculty of Medicine, Istanbul University, Turkey

## Abstract

**Background:**

Enthesitis-related arthritis (ERA) accounts for ∼10% to 20% of juvenile idiopathic arthritis (JIA) cases. Application of adult data to children with ERA is not ideal as less severe spinal involvement and more pronounced enthesitis and peripheral arthritis are present in children. Therefore, it is very important to conduct research that focuses specifically on the ERA population.

**Objectives:**

This study aimed to present an ERA inception cohort and determine which entheses and joints are most commonly affected, course of the disease and treatment approaches.

**Methods:**

Medical records of 546 patients with JIA who met each of the International League of Associations for Rheumatology (ILAR) criteria were evaluated retrospectively. An inception cohort of children with ERA who were diagnosed at Istanbul Faculty of Medicine between December 2020 and April 2022 was included. Patient characteristics were summarized by median and interquartile range (IQR) for continuous variables and frequency and percentage for categorical variables. *P* values <0.05 were considered statistically significant. All analyses were performed using SPSS statistical software version 28.

**Results:**

There were 34 newly diagnosed ERA patients. Sixty percent were male, and the median age at the onset of the disease was 11.5 years (interquartile range [IQR] 9–14.8 years). The majority of the group met the ILAR criteria for a diagnosis of ERA at the first pediatric rheumatology visit (*n* = 29 [85.2%]), with 5 subjects (14.7%) meeting the criteria at the second visit. Twenty-three (69 %) subjects had enthesitis and arthritis at the time of diagnosis. The median number of tender entheses at presentation was 2 (IQR 0–4), and 20 subjects (58%) had at least 1 tender enthesis. The most frequent enthesitis were located on the patellar ligament insertion at the inferior pole of the patella, the plantar fascial insertion at the calcaneus, the Achilles tendon insertion at the calcaneus, and the plantar fascial insertion at the metatarsal heads. Fifty-five (19)% of patients had symmetric enthesitis. The most commonly affected joints were the sacroiliacs, knees, and ankles. Medication use varied significantly across sites for children with peripheral arthritis (*p*< 0.001), but not for sacroiliitis or enthesitis only. Nonsteroidal anti-inflammatory drugs and disease-modifying antirheumatic drugs were the most commonly prescribed treatments, with anti-TNF agents primarily being initiation for sacroiliitis. HLA-B27 positivity was associated with male sex, higher active joint count, sacroiliitis, and higher disease activity at disease onset. The Juvenile Spondyloarthritis Disease Activity Index (JSpADA) was significantly higher in those who were HLA-B27-positive than in those who were HLA-B27-negative (*p* = 0.03). Those who were HLA-B27-positive were more likely to develop arthritis after the age of 6 years (*p*< 0.01).

**Conclusion:**

Either the observed heterogeneity in clinical presentation may result from differences in patient populations or from differences in the way ILAR criteria are applied. Most children have a pauciarticular onset and lower extremity enthesitis is common when ERA diagnosed.

